# Investigation and analysis of etiology associated with porcine respiratory disease complex in China from 2017 to 2021

**DOI:** 10.3389/fvets.2022.960033

**Published:** 2022-10-11

**Authors:** Qi Sun, Xuexiang Yu, Dongxian He, Xugang Ku, Bo Hong, Wei Zeng, Haifeng Zhang, Qigai He

**Affiliations:** ^1^State Key Laboratory of Agricultural Microbiology, College of Veterinary Medicine, Huazhong Agricultural University, Wuhan, China; ^2^The Cooperative Innovation Center for Sustainable Pig Production, Wuhan, China; ^3^Key Laboratory of Development of Veterinary Diagnostic Products, Ministry of Agriculture and Rural Affairs of the People's Republic of China, Wuhan, China; ^4^College of Animal Science and Technology, Guangxi Agriculural Vocational and Technical University, Nanning, China; ^5^Wuhan Green Giant Agriculture, Agriculture and Animal Husbandry Co., Ltd, Wuhan, China

**Keywords:** porcine respiratory diseases complex (PRDC), porcine reproductive and respiratory syndrome virus, *Streptococcus suis*, *Glaesserella (Haemophilus) parasuis*, prevalence characteristics, serotypes, coinfection

## Abstract

Porcine respiratory diseases complex (PRDC) is a highly serious threat to the pig industry. In the present study, we investigated and analyzed the etiology associated with PRDC and explored the role of viruses in respiratory bacterial infections. From 2017 to 2021, clinical samples were collected from 1,307 pigs with typical respiratory symptoms in 269 farms in China and screened for pathogens related to PRDC by PCR and bacterial isolation. The results indicated that PRRSV (41.16%, 95%CI: 38.49~43.83%), PCV2 (21.58%,95%CI: 19.35~23.81%), *S. suis* (63.50%, 95%CI: 60.89~66.11%), and *G. parasuis* (28.54%, 95%CI: 26.09~30.99%) were the most commonly detected pathogens in pigs with PRDC in China. The dominant epidemic serotypes (serogroups) of *S. suis, G. parasuis*, and *P. multocida* were serotype 2, serotype 1, and capsular serogroups D, respectively. Pigs of different ages exhibited different susceptibilities to these pathogens, e.g., PRRSV, PCV2, and *G. parasuis* had the highest detection rates in nursery pigs, whereas fattening pigs had the highest detection rates of *P. multocida* and *A. pleuropneumoniae*. Among the 1,307 pigs, the detection rates of *S. suis, G. parasuis, P. multocida*, and *B. bronchiseptica* were higher in virus-positive pigs, especially *G. parasuis* and *P. multocida* were significantly (*p* < 0.01) higher than in virus-negative pigs. In addition, a strong positive correlation was found between coinfection by PRRSV and *G. parasuis* (OR = 2.33, 95%CI: 1.12~2.14), PRRSV and *P. multocida* (OR = 1.55, 95%CI: 1.12~2.14), PCV2 and *P. multocida* (OR = 2.27, 95%CI: 1.33~3.87), PRRSV-PCV2 and *S. suis* (OR = 1.83, 95%CI: 1.29~2.60), PRRSV-PCV2 and *G. parasuis* (OR = 3.39, 95%CI: 2.42~4.74), and PRRSV-PCV2 and *P. multocida* (OR = 2.09, 95%CI: 1.46~3.00). In summary, PRRSV, PCV2, *S. suis*, and *G. parasuis* were the major pathogens in pigs with PRDC, and coinfections of two or more PRDC-related pathogens with strong positive correlations were common in China, such as PRRSV and *G. parasuis*, PRRSV and *P. multocida*, PCV2 and *P. multocida*, and also PRRSV-PCV2 and *G. parasuis* and PRRSV-PCV2 and *P. multocida*.

## Introduction

Porcine respiratory diseases complex (PRDC) is a general term for respiratory diseases caused by a combination of several factors such as viruses, bacteria, environmental conditions, and management strategies (1). It has emerged as a highly serious threat to the pig industry as it can result in various problems such as poor growth rate, poor feed conversion ratio, and severe acute death. Although PRDC has been reported to largely occur in 13~20-week-old fattening pigs, 5~12-week-old nursery pigs can also be affected, with the morbidity and mortality rate ranging from 30 to 70% and 4 to 6%, respectively (2, 3). The macroscopic lesions associated with PRDC are complex and often depend on the pathogen involved (1). Currently, the pathogens causing PRDC include viruses such as porcine reproductive and respiratory syndrome virus (PRRSV), porcine circovirus type 2 (PCV2), pseudorabies virus (PRV), and bacteria such as *Streptococcus suis* (*S. suis*); *Glaesserella (Haemophilus) parasuis* (*G. parasuis*); *Actinobacillus pleuropneumoniae* (*A. pleuropneumoniae*); *Pasteurella multocida* (*P. multocida*) and *Bordetella bronchiseptica* (*B. bronchiseptica*). Although each pathogen can cause a disease alone, simultaneous infection with the mentioned pathogens can often result in more severe clinical symptoms and lesions (4, 5). Numerous studies have assessed the prevalence of pathogens associated with respiratory diseases. For instance, in the USA, PRRSV (35.4%), *P. multocida* (31.6%), *M. hyopneumoniae* (27%), and swIAV (22.2%) were the most common pathogens, and mixed infections can be found in 88.2% of cases (6). In eastern China, PCV2 (59.9%), PRRSV (27.2%), *S. suis* (52.3%), and *G. parasuis* (33.2%) were the most common pathogens in clinically healthy pigs. In addition, coinfection was frequently detected, posing a great threat to the health of herds (7, 8). However, only a few studies have been conducted on the etiology associated with PRDC in diseased pigs in China.

PRRSV, PCV2, and PRV are regarded as the major pathogens of PRDC. These viruses can induce severe lesions and inhibit host immune functions in the respiratory system, thereby providing opportunities for invasion by secondary infectious pathogens (9–12). Bacteria are common secondary infection pathogens in commercial pig farms. Certain highly pathogenic bacteria such as *A. pleuropneumoniae, P. multocida*, and *B. bronchiseptica* can also cause primary infection and are the primary culprits causing acute death (13–15). At present, numerous studies have reported the interaction between viruses and bacteria such as PRRSV and *S. suis* (9, 10), PRRSV and *G. parasuis* (11), and PRV and *A. pleuropneumoniae* (12), indicating that immunosuppressive pathogens can extremely increase the susceptibility to bacterial infection, and coinfection can significantly increase the pathogenicity of bacteria in pigs (5). However, little scientific and clinical evidence exist to support this conclusion at the clinical level (7, 16, 17). Therefore, there is an urgent requirement to investigate and understand the correlation between immunosuppressive viruses and respiratory bacteria at the clinical level.

This study investigated the prevalence and coinfection characteristics of PRDC-related pathogens and analyzed the etiology associated with PRDC in diseased pigs in China. Furthermore, the relationship between viruses and bacteria of PRDC was analyzed at the clinical level. We believe the results of the study will help us understand the prevalent status of PRDC-related pathogens in China and provide clues for the prevention and control of PRDC.

## Materials and methods

### Sample collection

From 2017 to 2021, clinical samples were collected from 1,307 pigs with typical respiratory symptoms from 269 farms in 17 provinces (Table 1). Among them, samples were used for viral nucleic acid detection and bacterial isolation and identification. All samples were sent to the laboratory, followed by a sterile procedure to prevent cross-contamination, and bacterial isolation was conducted immediately. All procedures regarding animal care and testing were performed according to the recommendation of Hubei provincial public service facilities.

**Table 1:** Sample information.

**Years**	**Number of farms**	**Number of pigs**
2017	59	624
2018	52	211
2019	38	139
2020	35	111
2021	85	222
Total	269	1,307

### Viral nucleic acid detection

For viruses, tissue samples (lungs, inguinal lymph nodes, tonsils, etc. from the same pig were pooled) were homogenized using TissueLyser-II (QIAGEN, Germany). Afterward, DNA and RNA were extracted using the TIANamp Virus DNA/RNA Kit (TIANGEN, Beijing, China) according to the manufacturer's instructions. PRRSV, PCV2, and PRV were detected by PCR methods, and the primer sequences used are listed in Supplementary Table S1. Primers were synthesized by Sangon Biotech Co., Ltd (Shanghai). The extracted DNA and RNA were stored at −80°C.

### Culture conditions and identification methods

Five common respiratory bacteria, namely, *S. suis, G. parasuis, P. multocida, A. pleuropneumoniae, P. multocida*, and *B. bronchiseptica* were isolated and identified from samples (namely, nasal swabs, trachea, lungs, spleen, brain and joint fluid), using Tryptic Soy Broth (TSB) or Tryptic Soy Agar (TSA) (Difco Laboratories, Detroit, USA) with 10 μg/ml of nicotinamide adenine dinucleotide (NAD) and 10% (v/v) inactivated cattle serum (Zhejiang Tianhang Biotechnology, Zhejiang, China). All plates were incubated at 37°C for 24 to 48 h, followed by the purification of these strains by colony morphology, Gram-staining, and oxidase status (Gram-negative bacilli) or catalase tests. To further confirm the phenotypic and biochemical results, PCR methods were used (18–22), and the primer sequences used are listed in Supplementary Table S1. Primers were synthesized by Sangon Biotech Co., Ltd (Shanghai). All isolated bacteria were kept at −80°C.

### Serotype identification of *S. suis, H. parasuis*, and *P. multocida*

Based on previous reports, *S. suis* is divided into 33 serotypes according to the difference in its capsular polysaccharide. In this study, we only focused on serotypes 1 to 10 and used the first multiplex PCR (mPCR) assay (serotypes 1 to 10, 1/2, and 14) developed by Liu (23). To further identify serotypes 1 and 14, 2, and 1/2, the PCR assay developed by Thu was used (24). *G. parasuis* can be classified into 15 serotypes according to the differences in its capsular loci. We used a molecular serotyping PCR and a rapid PCR to identify the serotypes of *G. parasuis*. The mPCR was developed by Howell, which can discriminate between all serotypes of *G. parasuis* except serotypes 5 and 12 (25), and the PCR assay developed by Jia was used to further identify serotypes 5 and 12 (26). *P. multocida* can be classified into five capsular serogroups based on the difference in capsular biosynthetic loci. The PCR assay developed by Townsend was used to identify capsular serogroups A, B, D, E, and F of *P. multocida* (27).

### Statistical analysis

At the farm and individual sample levels, all research data were analyzed. Multiple samples were simultaneously used for bacterial isolation from the same pig. As long as a certain bacterium was isolated in one of the samples, the pig was judged to be positive for this bacterium. The prevalence of each pathogen was calculated, and the binomial exact method was used to compute 95% confidence intervals (CIs). Univariate association between detection rates of different viruses and isolation rates of different bacteria was determined using the univariate ordinary logistic regression analysis, Chi-square test (χ^2^), Phi index, and Fisher's exact test. *p* < 0.05 and *p* < 0.01 were considered significant and highly significant, respectively. The SPSSAU (version 22.0) data scientific analysis platform (https://www.spssau.com) was used for data analysis.

## Results

### Prevalence characteristics of major pathogens

**Figure 1:**
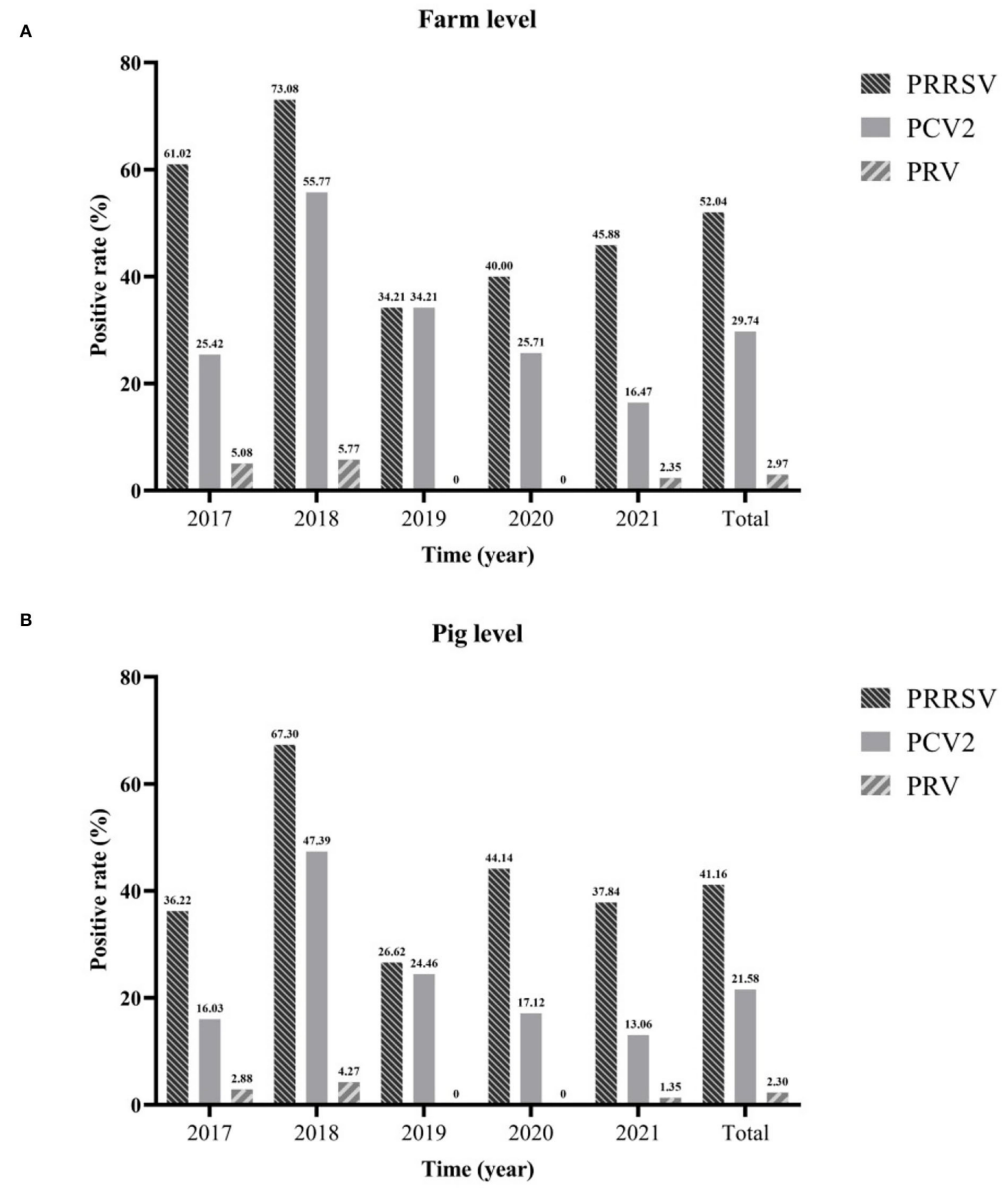
The positive rate of different viruses from 2017 to 2021. Farm level **(A)** and pig level **(B)**.

In this study, 1,307 samples from 2017 to 2021 were collected from 269 pig farms for viral nucleic acid detection. These results showed that the positive rates of farm level and pig level of PRRSV were the highest, which were 52.04% (95%CI: 46.07~58.01%) and 41.16% (95%CI: 38.49~43.83%), respectively. This was followed by PCV2, the positive rates of farm level and pig level were 29.74% (95%CI: 24.28~35.20%) and 21.58% (95%CI: 19.35~23.81%). The positive rate of PRV was the lowest, only 2.97% (95%CI: 0.94~5.00%) and 2.30% (95%CI: 1.48~3.11%), respectively. Regardless of the farm or pig level, PRRSV, PCV2, and PRV had the highest positive rates in 2018 [farm level: PRRSV (73.08%, 95%CI: 61.02~85.13%), PCV2 (55.77%, 95%CI: 42.27~69.27%) and PRV (5.77%, 95%CI: 0.00~12.11%); pig level: PRRSV (67.30%, 95%CI: 60.97~73.63%), PCV2 (47.39%, 95%CI: 40.66~54.13%) and PRV (4.27%, 95%CI: 1.54~6.99%)]. Over the past 5 years, the positive rate of PRRSV has remained high, with the lowest in 2019 [farms: 34.21% (95%CI: 19.13~49.29%) and pigs: 26.62% (95%CI: 19.27~33.97%)] and followed by an annual increase until 2021. Although PCV2 has been declining yearly since 2018, the positive rates of farms and pigs in 2021 were the lowest, 16.47% (95%CI: 8.59~24.36%) and 13.06% (95%CI: 8.63~17.50%), respectively. The positive rate of PRV was maintained at a low level, i.e., 0.00% in both 2019 and 2020 (Figure 1).

**Figure 2:**
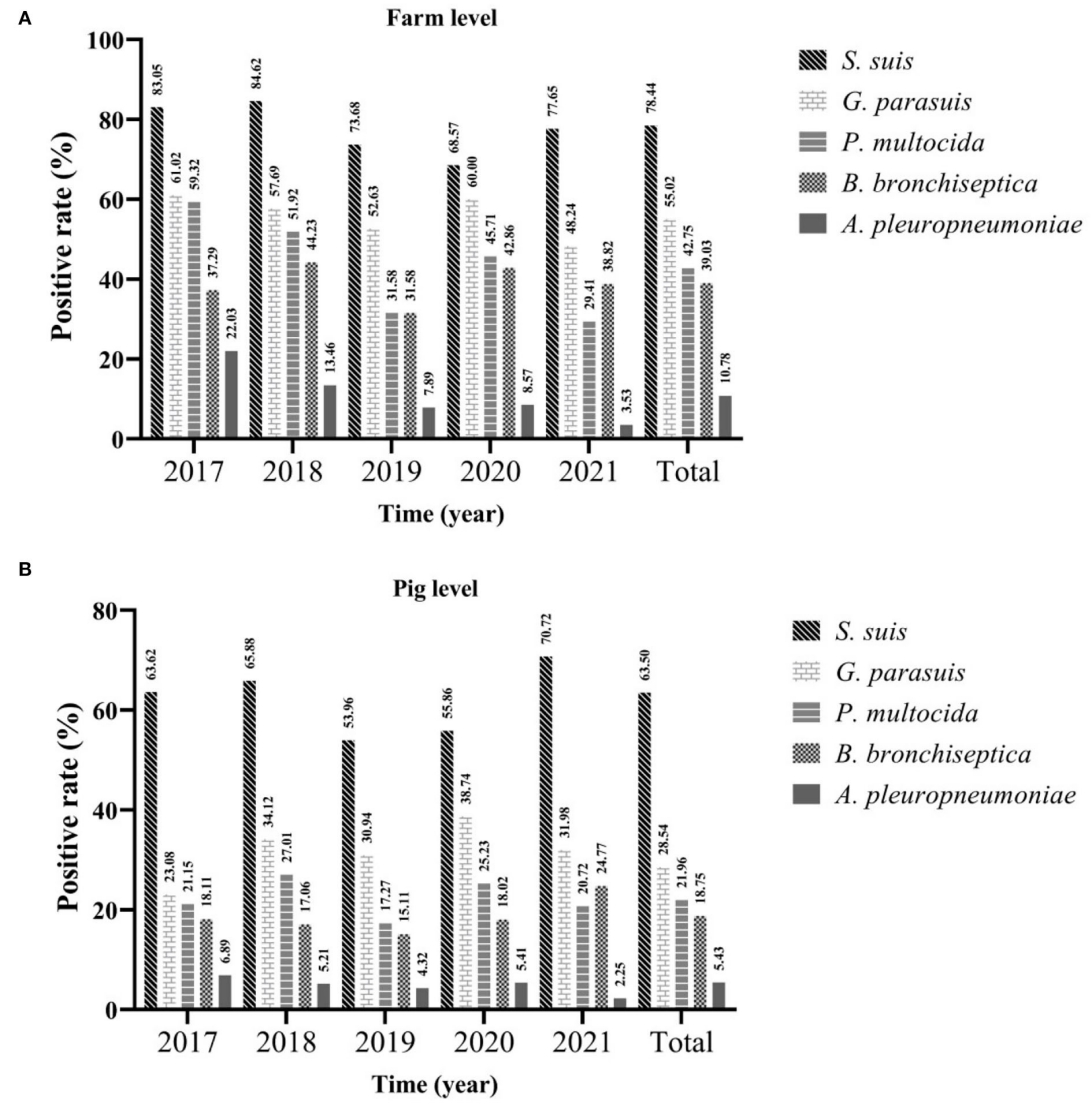
The positive rate of different bacteria from 2017 to 2021. Farm level **(A)** and pig level **(B)**.

In this study, bacteria isolation and identification were performed on samples of 1,307 diseased pigs in 269 farms collected between 2017 and 2021. The statistics showed that the positive rates of farm level and pig level of *S. suis* were the highest, which were 78.44% (95%CI: 73.52~83.35%) and 63.50% (95%CI: 60.89~66.11%), respectively, followed by *G. parasuis*, whose positive rate of farms was 5.02% (95%CI: 49.07~60.96%) and the positive rate of pigs was 28.54% (95%CI: 26.09~30.99%). The positive rate of *A. pleuropneumoniae* was the lowest, only 10.78% (95%CI: 7.07~14.49%) and 5.43% (95%CI: 4.20~6.66%), respectively. Regardless of the farm or pig level, the positive rate of *S. suis* had been consistently high [farm level ranged from 68.57% (95%CI: 53.19~83.95%) to 83.05% (95%CI: 73.48~92.62%); pig level ranged from 53.69% (95%CI: 45.67~62.24%) to 70.72% (95%CI: 64.73~76.71%)], followed by *G. parasuis, P. multocida*, and *B. bronchiseptica*. Although in the past 5 years, the positive rate of these bacteria has fluctuated, it has largely remained stable, and the positive rate of *B. bronchiseptica* surpassed that of *P. multocida* for the first time in 2021. Although the positive rate of *A. pleuropneumoniae* has remained low, and has declined yearly since 2017 [farm level ranged from 22.03% (95%CI: 11.46~32.61%) to 3.53% (95%CI: 0.00%~7.45%); the pig level ranged from 6.89% (95%CI: 4.90~8.88%) to 2.25% (95%CI: 0.30~4.20%)] (Figure 2).

### Serotype identification of *S. suis, G. parasuis*, and *P. multocida*

**Figure 3:**
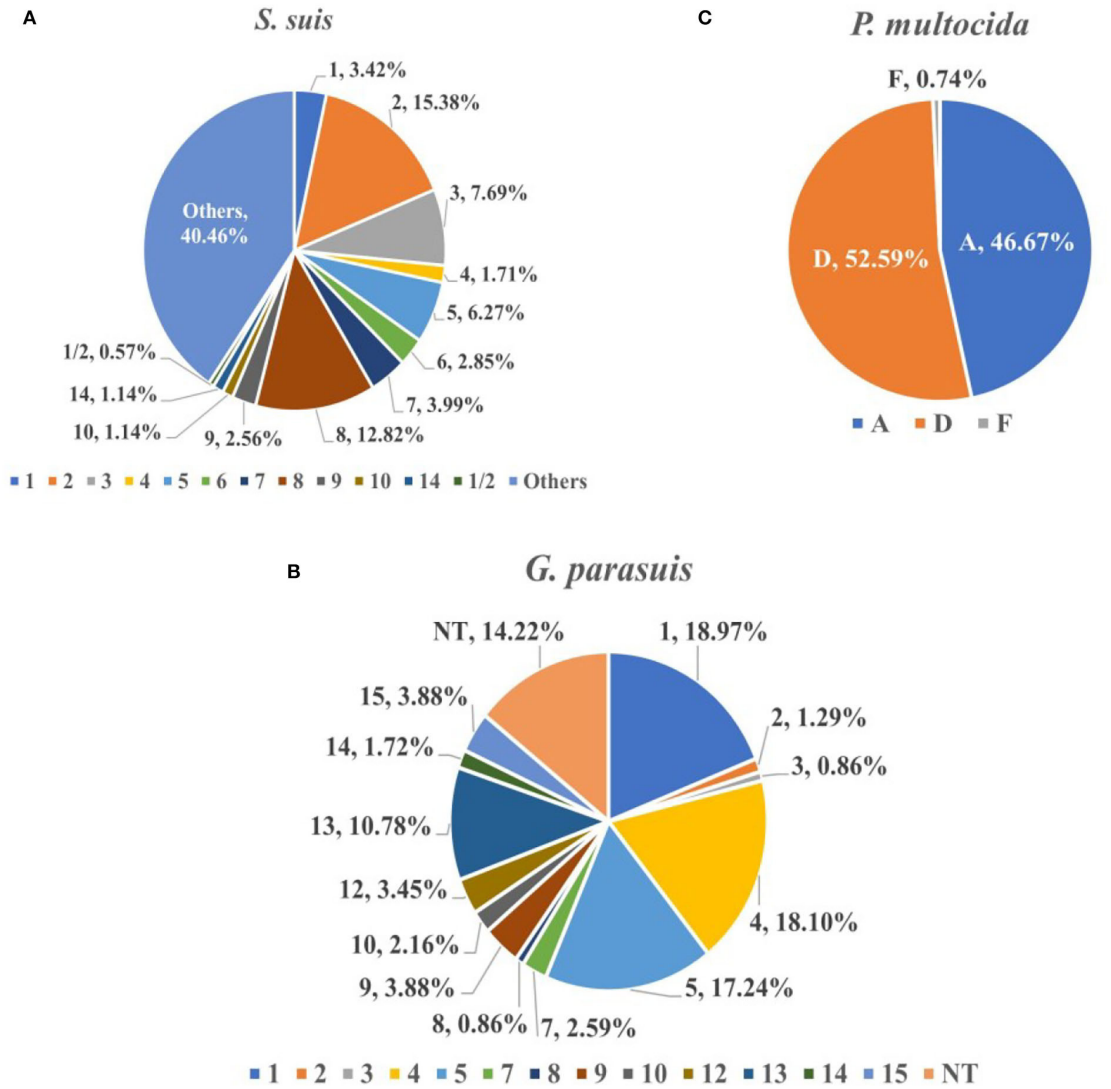
The proportion of different serovars (serogroups) of *S. suis* **(A)**, *G. parasuis* **(B)**, and *P. multocida* **(C)**. *S. suis* (*n* = 351), *G. parasuis* (*n* = 232) and *P. multocida* (*n* = 135).

From 2017 to 2021, the serotype identification of three bacteria with higher isolation rates was performed, and strains of the same serotype detected on the same farm were counted only once. The serotype identification results of 351 *S. suis* strains showed that the highest proportion was that of virulent strain serotype 2 (15.38%, 95%CI: 11.61~19.16%), followed by the moderately virulent serotype 8 (12.82%, 95%CI: 9.32~16.32%) and serotype 3 (7.69%, 95%CI: 4.90~10.48%). The recognized virulent strains (serotypes 1, 2, 7, and 9) accounted for 25.36% (89/351, 95%CI: 20.80~29.91%) (Figure 3A). Although the detection rate of most serotypes has remained relatively stable during the last 5 years, that of serotype 9 has been decreasing yearly, and the proportion in 2020 and 2021 was the same (0.00%). In addition, serotype 1/2 strains were isolated in 2018 and 2021, proving that serotype 1/2 infection exists in China (0.57%, 95%CI: 0.00~1.36%). For *G. parasuis* (*n* = 232), the highest proportion was recorded for virulent strain serotype 1 (18.97%, 95% CI: 13.92~24.01%), followed by the moderately virulent serotype 4 (18.10%, 95%CI: 13.15~23.06%), and virulent strains serotype 5 (17.24%, 95%CI: 12.38~22.10%), non-typeable (NT) strains accounted for 14.22% (95%CI: 9.73~18.72%) (Figure 3B). The increase in the proportion of serotype 1 occurred primarily in 2021, from 16.90% on average to 26.79% by 2020. In contrast, the prevalence of the previous mainstream serotype 5 decreased from 25.37% in 2017 to 12.50% in 2021. In addition, we identified the capsular serogroups of 135 *P. multocida*, and no strains of capsular serogroups B and E were found. The highest proportion was of capsular serogroup D (52.59%, 95%CI: 44.17~61.02%), followed by capsular serogroup A (46.67%, 95%CI: 38.25~55.08%), and only one capsular serogroup F strain was isolated in 2019 (0.74%, 95% CI: 0.00~2.19%) (Figure 3C).

### Seasonal prevalence characteristics of major pathogens

**Figure 4:**
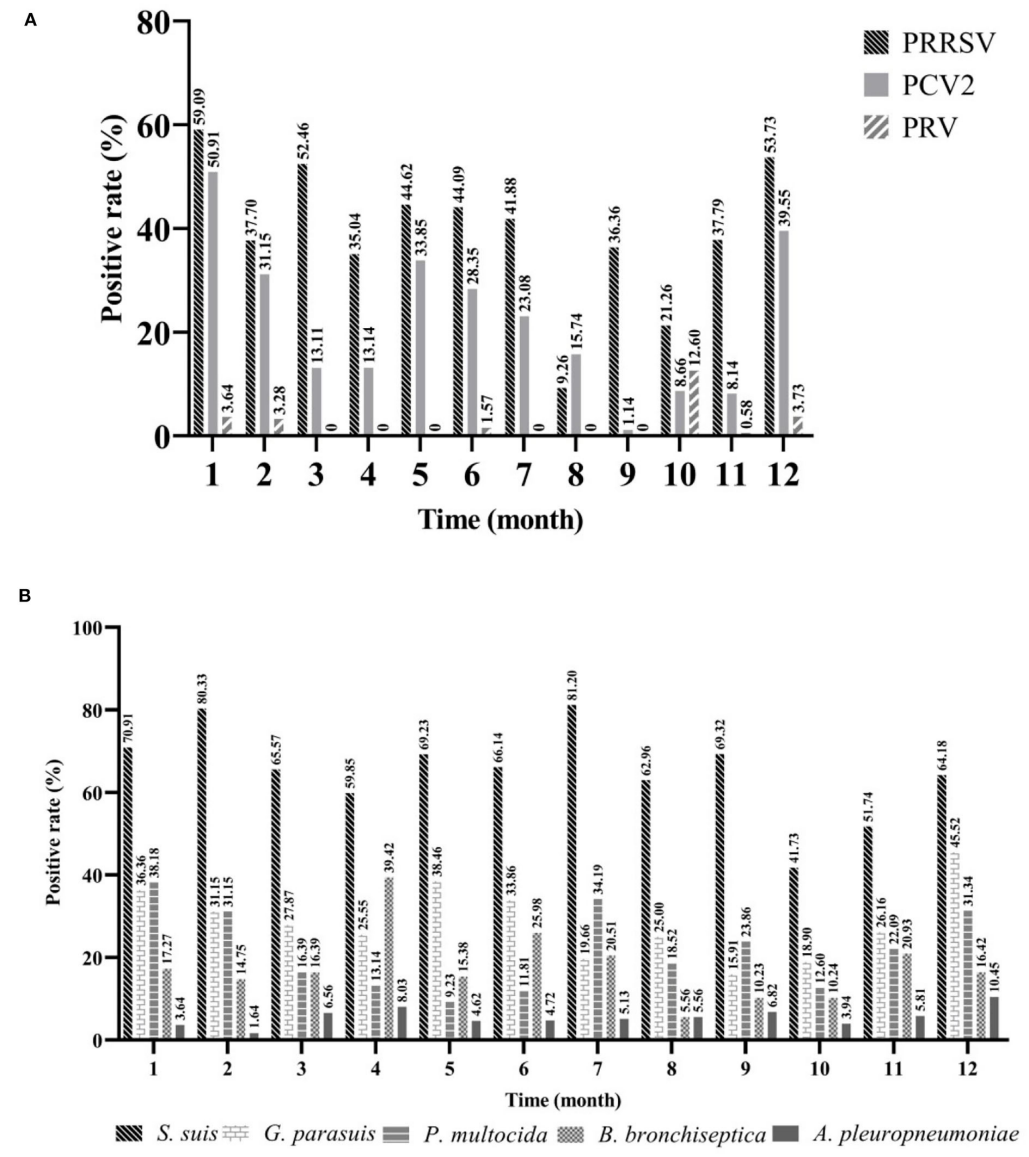
The seasonal prevalence characteristics of major pathogens. Viruses **(A)** and bacteria **(B)**. Jan (*n* = 110), Feb (*n* = 61), Mar (*n* = 61), Apr (*n* = 137), May (*n* = 65), Jun (*n* = 127), Jul (*n* = 117), Aug (*n* = 108), Sep (*n* = 88), Oct (*n* = 127), Nov (*n* = 172), Dec (*n* = 134).

To explore the prevalence of viruses in different months, positive rates were counted on a monthly basis. The detection rate of PRRSV was the highest in January (59.09%, 95%CI: 32.24~39.49%) and the lowest in August (9.26%, 95%CI: 2.79~14.73%). The positive rate of PRRSV in winter (December to February) (52.46%, 95%CI: 46.85~58.06%) was extremely (*p* < 0.001) higher than that in summer (June to August) (32.67%, 95%CI: 27.77~37.57%) (χ^2^ = 26.29). Similar to PRRSV, PCV2 had the highest detection rate in January (50.91%, 95%CI: 41.57~60.25%); however, the lowest detection rate was recorded in September (1.14%, 95%CI: 0.00~3.35%), and the detection rate of PCV2 was extremely (*p* < 0.001) higher in winter (41.97%, 95%CI: 36.43~47.51%) than in autumn (September to November) (6.72%, 95%CI: 4.22~9.21%) (χ^2^ = 122.49). The prevalence of PRV in this study was at a low level, with the highest detection rate in October (12.60%, 95%CI: 6.83~18.37%) (Figure 4A). It indicated that more attention should be paid to the prevention and control of viral diseases in cold environments.

To explore the prevalence of bacteria in different months, isolation rates were calculated on a monthly basis. The isolation rate of *S. suis* was higher than that of other bacteria every month, and the highest was recorded in July (81.20%, 95%CI: 74.12~88.28%), followed by February (80.33%, 95%CI: 70.35~90.30%), and lowest in October (41.73%, 95%CI: 33.16~50.31%). Next, *G. parasuis* had the highest isolation rate in December (45.52%, 95%CI: 37.09~53.95%), followed by May (38.46%, 95%CI: 26.63~50.29%), and lowest in September (15.91%, 95%CI: 8.27~23.55%). For *P. multocida*, its isolation rate was the highest in January (38.18%, 95%CI: 29.10~47.26%), followed by July (34.19%, 95%CI: 25.59~47.26%), and lowest in May (9.23%, 95%CI: 2.19~16.27%). The prevalence of *B. bronchiseptica* was the highest in April (39.42%, 95%CI: 31.23~47.60%), followed by June (25.98%, 95%CI: 18.36~33.61%), and lowest in August (5.56%, 95%CI: 1.24~9.88%). The isolation rate of *A. pleuropneumoniae* was consistently low, highest in December (10.45%, 95%CI: 5.27~15.63%), and lowest in February (1.64%, 95%CI: 0.00~4.83%) (Figure 4B). Compared to these viruses, bacteria appeared to show higher isolation rates during both summer and winter.

### Prevalence characteristics of major pathogens among different stages

**Figure 5:**
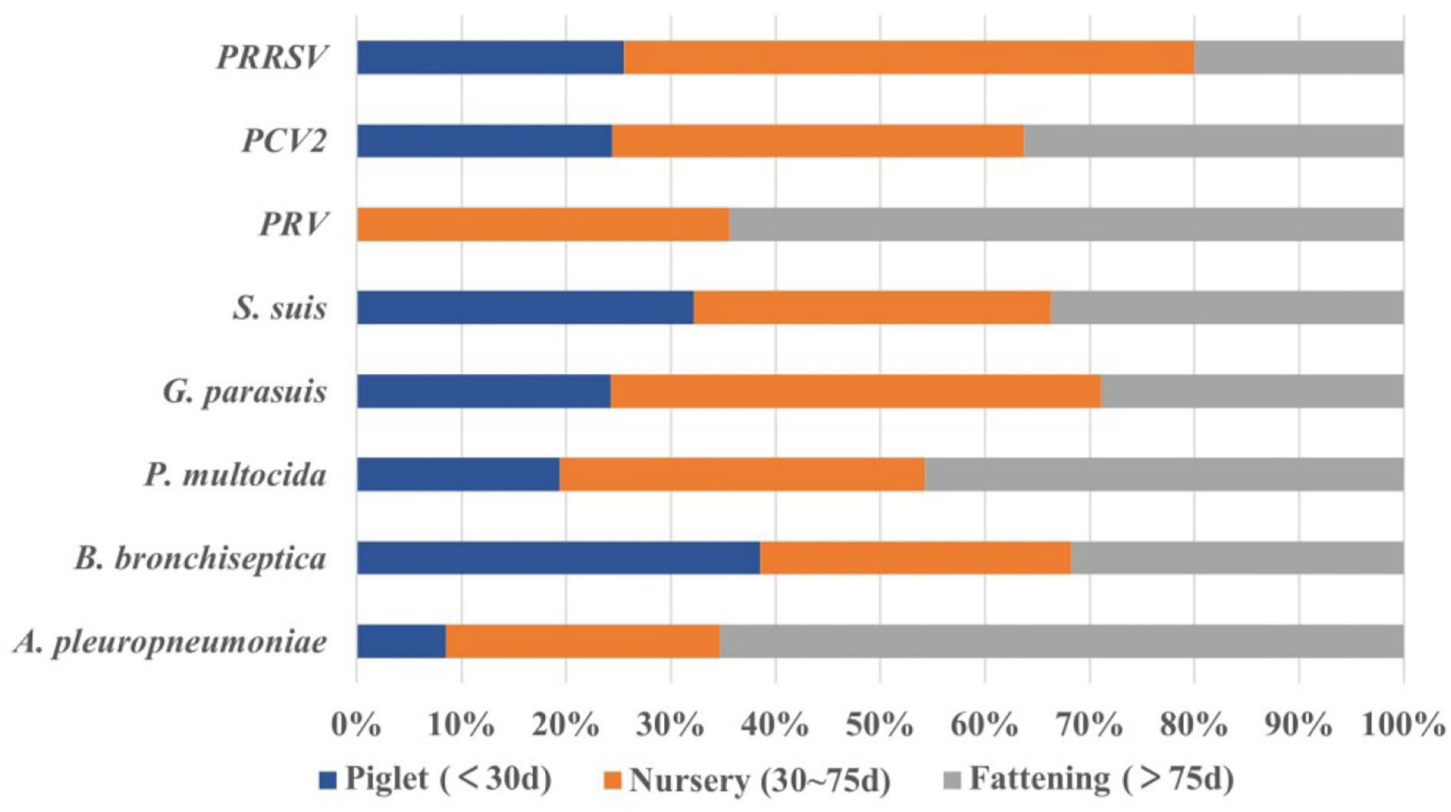
The proportion of pathogens among different stages (age). Piglets (*n* = 91), nursery pigs (*n* = 622), and fattening pigs (*n* = 594).

A total of 1,307 pigs collected from 2017 to 2021 were divided into farrowing piglets (< 30 days) (*n* = 91), nursery pigs (30~75 days) (*n* = 622), and fattening pigs (>75 days) (*n* = 594) according to their age. We found that pigs with respiratory symptoms were largely concentrated in the nursery (47.59%, 622/1,307) and fattening pigs (45.45%, 594/1,307). The positive rate of PRRSV in nursery pigs was the highest (60.93%, 95%CI: 57.10~64.77%), and was significantly higher (*p* < 0.001) than that in piglets (χ^2^ = 32.58) and fattening pigs (χ^2^ = 183.58). Similarly, PCV2 had the highest positive rate in nursery pigs (22.99%, 95%CI: 19.68~26.30%), and was significantly higher (*p* < 0.001) than that in piglets (χ^2^ = 67.91). PRV had the highest positive rate in fattening pigs (3.20%, 95%CI: 1.78~4.61%); however, there was no significant difference between the other two groups. For bacteria, the isolation rate of *S. suis* at all stages was over 60%, among which nursery pigs showed the highest isolation rate (64.15%, 95%CI: 60.38~67.92%); however, there was no significant difference between the other two groups. The isolation rate of *G. parasuis* in nursery pigs was as high as 36.01% (95%CI: 32.24~39.49%), which was significantly higher than the isolation rate recorded in piglets (χ^2^ = 9.90) and fattening pigs (χ^2^ = 27.25). Both *P. multocida* and *A. pleuropneumoniae* had the highest isolation rates in fattening pigs (25.93% (95%CI: 22.40~29.45%) and 8.42% (95%CI: 6.18~10.65%), respectively), Among them, the isolation rate of *P. multocida* in fattening pigs was extremely and significantly (*p* < 0.01) higher than that in piglets (χ^2^ = 8.87), and significantly (*p* < 0.05) higher than that in the nursery stage (χ^2^ = 6.19). The isolation rate of *A. pleuropneumoniae* in fattening pigs was extremely and significantly (*p* < 0.001) higher than that in the nursery stage (χ^2^ = 13.14), and significantly (*p* < 0.05) higher than that in piglets (χ^2^ = 5.12). Finally, we found that *B. bronchiseptica* had the highest isolation rate in piglets (23.08%, 95%CI: 14.42~31.73%) with no significant difference from the other two groups (Figure 5). These results showed that pigs of different ages had different susceptibilities to these pathogens, and we should focus on the corresponding pathogens for each stage of pigs.

### Prevalence characteristics and correlations of coinfection between pathogens

At present, mixed infection is highly common in China. To explore the impact of viruses on bacterial infection under clinical conditions, pigs infected with different viruses were grouped and compared with virus-negative groups [virus (-)] (51.80%, 95%CI: 49.09~54.51%). For PRRSV, 326 pigs (24.94%, 95%CI: 22.60~27.29%) were infected with PRRSV only (negative for PCV2 and PRV) in 1,307 samples. As can be seen from Table 2, the isolation rate of *S. suis, G. parasuis, P. multocida*, and *B. bronchiseptica* was higher in PRRSV (+). It is apparent from Table 3 that the isolation rate of *G. parasuis* was 36.50% (95%CI: 31.28~41.73%), which was extremely (*p* < 0.001) higher than that of the virus (-) (χ^2^ = 31.69). The isolation rate of *P. multocida* (24.23%, 95%CI: 19.58~28.88%) was significantly (*p* < 0.05) higher than that of the virus (-) (χ2 = 6.63); whereas there was no significant difference among *S. suis, B. bronchiseptica*, and *A. pleuropneumoniae*. Furthermore, the probability of the detection of *G. parasuis* and *P. multocida* was 2.33 (OR, 95%CI: 1.12~2.14) and 1.55 (OR, 95%CI: 1.12~2.14) times more likely in PRRSV (+) than in virus-negative pigs. For PCV2, 72 pigs were infected with PCV2 only (5.51%, 95%CI: 4.27~6.75%) in 1,307 samples. The statistical results showed that the isolation rate of *G. parasuis* and *P. multocida* was higher in PCV2 (+), and the isolation rate of *P. multocida* was 36.50% (95%CI: 21.17~42.71%) significantly (*p* < 0.01) higher than that of the virus (-) (χ^2^ = 8.49), whereas there was no significant difference among *S. suis, G. parasuis, B. bronchiseptica*, and *A. pleuropneumoniae*. In addition, the probability of the detection of *P. multocida* was 2.27 (OR, 95%CI: 1.33~3.87) times more likely in PCV2 (+) than in virus-negative pigs (Tables 2, 3).

**Table 2:** Isolation rate of various bacteria under different virus infections.

	**PRRSV (+) (*n* = 326)**	**PCV2 (+)** ** (*n* = 72)**	**PRRSV-PCV2 (+) (*n* = 202)**	**Virus (+)** ** (*n* = 630)**	**Virus (-)** ** (*n* = 677)**
***S. suis***	**66.26%**	**50.00%**	**73.76%**	**66.67%**	**60.56%**
***G. parasuis***	**36.50%**	**23.61%**	**45.54%**	**37.94%**	**19.79%**
***P. multocida***	**24.23%**	**31.94%**	**30.20%**	**27.14%**	**17.13%**
***B. bronchiseptica***	**20.25%**	**12.50%**	**21.78%**	**19.37%**	**18.17%**
***A. pleuropneumoniae***	**3.68%**	**12.50%**	**3.47%**	**4.44%**	**6.50%**

PRRSV (+), positive for PRRSV only; PCV2 (+), positive for PCV2 only; PRRSV-PCV2 (+), positive for both PRRSV and PCV2; Virus (+), positive for at least one virus (PRRSV, PCV2, or PRV); Virus (-), negative for PRRSV, PCV2, and PRV.

**Table 3:** Descriptive statistics and odds ratios for the detection of various bacteria under different immunosuppressive virus infections (compared with virus-negative).

	**PRRSV (+)**		**PCV2 (+)**		**PRRSV-PCV2 (+)**		**Virus (+)**	
	**χ^2^**	**OR(95%CI)**	**χ^2^**	**OR(95%CI)**	**χ^2^**	**OR(95%CI)**
*S. suis*	2.81	1.28(0.97~1.69)	2.59	0.65(0.40~1.06)	11.15***	1.83(1.29~2.60)	4.99*	1.30(1.04~1.63)
*G. parasuis*	31.69***	2.33(1.74~3.13)	0.38	1.25(0.70~2.23)	52.67***	3.39(2.42~4.74)	51.79***	2.48(1.93~3.18)
*P. multocida*	6.63*	1.55(1.12~2.14)	8.49**	2.27(1.33~3.87)	15.71***	2.09(1.46~3.00)	18.50***	1.80(1.38~2.35)
*B. bronchiseptica*	0.49	1.14(0.82~1.60)	1.08	0.64(0.31~1.33)	1.1	1.25(0.85~1.85)	0.23	1.08(0.82~1.43)
*A. pleuropneumoniae*	2.8	0.55(0.29~1.06)	2.71	2.06(0.96~4.41)	2.09	0.52(0.23~1.17)	2.27	0.67(0.41~1.09)

*, ** and *** denote p-values of p < 0.05, p < 0.01 and p < 0.001, respectively. OR, odds ratio.

Previous studies have reported that coinfection with PRRSV and PCV2 can lead to more severe immunosuppression. Therefore, we compared the coinfected PRRSV and PCV2 group PRRSV-PCV2 (+) to virus (-). A total of 202 pigs were coinfected with PRRSV and PCV2 in 1,307 samples (24.94%, 95% CI: 22.60~27.29%). The results showed that the isolation rate of *S. suis, G. parasuis, P. multocida*, and *B. bronchiseptica* was higher in PRRSV-PCV2 (+), and the isolation rate of *S. suis* (73.76%, 95%CI: 67.70~79.83%), *G. parasuis* (45.54%, 95%CI: 38.68~ 52.41%), and *P. multocida* (30.20%, 95%CI: 23.87~36.53%) dramatically (*p* < 0.001) higher than these [60.56% (χ^2^=11.15), 19.79% (χ^2^=52.67), and 17.13% (χ^2^=15.71)] in the virus (-), whereas there was no significant difference between these two groups for *B. bronchiseptica* and *A. pleuropneumoniae*. Moreover, the probability of the detection of *S. suis, G. parasuis*, and *P. multocida* was 1.83 (OR, 95%CI: 1.29~2.60), 2.33 (OR, 95%CI: 1.12~2.14), and 1.55 (95%CI: 1.12~2.14) times more likely in PRRSV-PCV2 (+) than in virus-negative pigs (Tables 2, 3). The probability of finding *S. suis, G. parasuis*, and *P. multocida* in coinfection with at least one virus (PRRSV, PCV2, or PRV) was more than 1.30 (OR, 95%CI: 1.04~1.63), 2.48 (OR, 95%CI: 1.93~3.18), and 1.80 (OR, 95%CI: 1.38~2.35) times higher than that in virus-negative pigs (Table 3). Therefore, these results demonstrate that immunosuppressive pathogens such as PRRSV and PCV2 increased the isolation rate of *S. suis, G. parasuis*, and *P. multocida*. In contrast, the isolation rate of virulent strains such as *A. pleuropneumoniae* and *B. bronchiseptica* did not appear to be affected by these viruses.

## Discussion

Porcine respiratory diseases complex is one of the important problems that has plagued the development of the pig industry, causing great economic losses. Pathogens such as PRRSV, PCV2, and *A. pleuropneumoniae* have been implicated in these diseases. Numerous studies have reported that immunosuppressive pathogens can significantly increase the risk of bacterial infections in animals and *in vitro* tests; however, these conclusions are rarely supported by scientific clinical data (5). Therefore, we investigated the prevalence and coinfection characteristics of PRDC-related pathogens and analyzed the etiology associated with PRDC in diseased pigs in China, and explored the effect of porcine respiratory viruses on the bacteria at the clinical level.

We found that among diseased pigs, the detection rate of PRRSV was the highest of these three viruses, which was consistent with the reports on the PRRSV epidemic in China in recent years (28–31). It indicated that PRRSV is still one of the high pathogens threatening the Chinese swine industry. The prevalence of PRRSV from 2017 to 2021 showed an initial decreasing trend, followed by an annual increase after reaching the lowest in 2019, which is also consistent with the latest research (32). PCV2 is another important disease that can lead to immunosuppression. Its positive rate has remained high and declined after the highest detection rate in 2018, causing great trouble to the Chinese pig industry (33–36). The positive rate of PRV in this study has been at a low level, with a clear downward trend since 2018. The possible reason is that the use of safe vaccines and effective differential diagnosis methods have resulted in a mature Chinese pig farm system for PRV prevention and control (37–40).

For bacteria, *S. suis* showed the highest positive rate (>50%) in diseased pigs with PRDC-related symptoms, followed by *G. parasuis* and *P. multocida*, whereas *A. pleuropneumoniae* was maintained at a lower level, which was consistent with the results of related studies in recent years (15, 41, 42). Between 2017 and 2021, no changes in the epidemic characteristics of these bacteria were observed, and the annual positive rate tended to be stable, whereas the positive rate of *A. pleuropneumoniae* declined annually since 2017 (42). It was demonstrated that the primary respiratory pathogens in China were still *S. suis, G. parasuis*, and *P. multocida*. Although these pathogens are often regarded as secondary pathogens in PRDC, highly virulent strains can still cause serious symptoms.

Several bacteria have multiple serotypes with different pathogenicity and lack of cross-protection, posing serious problems with the selection of vaccines for bacterial diseases (23, 25). Therefore, the serotypes of *S. suis, G. parasuis*, and *P. multocida*, which have the highest isolation rate, were identified. The results showed that the most prevalent *S. suis* in China was still serotype 2 with high virulence, followed by serotypes 8, 3, and 5, whereas the recognized virulent strains (serotypes 1, 2, 7, and 9) accounted for 25.36%. After introducing a new method, serotypes 1 and 14, 2, and 1/2 were re-investigated, and serotype 1/2 infection was found in China as early as 2018. Between 2017 and 2021, the detection rates of most serotypes were relatively stable, but the detection rate of serotype 9 decreased dramatically, which was considerably different from the latest survey results (17, 41, 42), suggesting a new change in the epidemic situation of *S. suis* in China. For *G. parasuis*, a similar phenomenon occurred in different serotypes. The results showed that the most prevalent *G. parasuis* serotypes in China were the virulent serotype 1, followed by serotypes 4, 5, and 13. Over the past 5 years, the detection rate of serotype 1 has shown a gradual increase, particularly in 2021, and the proportion increased significantly. In contrast, the detection rate of serotype 5, which was the highest proportion in the past, decreased annually, which undoubtedly showed that the serotype trend of *G. parasuis* in China is changing (17, 42, 43). For *P. multimedia*, the results showed that the most prevalent strains in China were still the capsular serogroups D strains, followed by the capsular serogroups A, which was consistent with the previous research results (44, 45). The development of bacterial vaccines is closely related to their serotypes, suggesting that monitoring bacterial serotypes is a long-term and significant task.

An analysis of the positive rates of those pathogens among different months revealed the characteristics of their seasonal distribution. The detection rate of PRRSV in winter was significantly higher (*p* < 0.001) than those in summer, which was contrary to the results of certain studies (31, 32). This is primarily because PRRSV can cause a multisystem disease and we only focused on the respiratory system. Similar to PRRSV, PCV2 had the highest detection rate in winter, whereas PRV showed a higher detection rate between October and February (37). Viruses can easily survive in cold environments, indicating that winter is still the season of a high incidence of viral diseases. For these five bacteria, we found a seasonal pattern, all showing higher isolation rates during summer and winter. This could be related to the ventilation frequency and the air quality of the pig house. Environmental and management conditions have been shown to be of major importance in the modulation of bacterial infection (17, 42).

The PRDC onset can be observed by dividing the stage of pigs, as well as, the primary pathogens. The results of this study demonstrated no significant difference in the detection rate of *S. suis* at different stages. However, these were higher than 60%, suggesting that we should pay attention to its prevention and control at every stage. PRRSV, PCV2, and *G. parasuis* were more common in nursery pigs, suggesting that we should increase our awareness of these three diseases at this stage. The detection rates of *P. multimedia* and *A. pleuropneumoniae* in fattening pigs were significantly higher than in other stages, indicating that these two bacteria as the primary cause of diseases in fattening pigs. *B. bronchiseptica* is also the primary pathogen causing necrohemorrhagic bronchopneumonia in young pigs, it may explain the highest isolation rate in piglets in this study (1, 5). It suggests that pigs of different ages have different susceptibilities to those pathogens, and we should focus on the corresponding pathogens for each stage of pigs.

In these diseased pigs, coinfection by multiple pathogens was highly common, and even six pathogens were detected in the same pig. PRRSV, PCV2, and PRV are widely recognized as the major pathogens of PRDC and can aggravate the severity of bacterial pneumonia (11, 12, 46). In this study, the impact of immunosuppressive pathogens on common respiratory bacteria was investigated at the clinical farm level. Among the 1,307 pigs, the detection rates of *S. suis, G. parasuis, P. multocida*, and *B. bronchiseptica* were higher in virus-positive pigs, especially detection rates of *G. parasuis* and *P. multocida* were significantly (*p* < 0.01) higher than those in virus-negative pigs. The probability of detecting *G. parasuis* and *P. multocida* in PRRSV-positive pigs was 2.33 and 1.55 times higher than in virus-negative pigs. The probability of detecting *P. multocida* in PCV2-positive pigs was 2.27 times higher than that in virus-negative pigs. It indicated that PRRSV could increase the chance of infection by *G. parasuis* and *P. multocida*, and PCV2 may increase the chance of infection by *P. multocida*. Previous studies have demonstrated that PRRSV-PCV2 coinfection can result in more severe immunosuppression. We found that the detection rates of *S. suis, G. parasuis*, and *P. multocida* in PRRSV-PCV2 (+) were dramatically (*p* < 0.01) higher than those in pathogen-negative pigs, and the probability of the detection of *S. suis, G. parasuis*, and *P. multocida* was 1.83, 3.39, and 2.09 times in PRRSV-PCV2 coinfected pigs than in virus-negative pigs, which was also higher than that in PRRSV-positive pigs and PCV2-positive pigs (1, 11, 16, 47, 48). It revealed that immunosuppressive viruses could increase the chance of certain bacterial (such as *G. parasuis* and *P. multocida*) infections. Conversely, no significant difference was observed in the detection rate of *A. pleuropneumoniae* and *B. bronchiseptica* between those groups. It implied that there were no correlations between coinfection by *A. pleuropneumoniae* (*B. bronchiseptica*) and those three viruses.

In addition, to exclude the influence of other factors, each sample was collected from pigs with respiratory symptoms. The same samples, if collected from healthy pigs, might yield different results. There are other pathogens in PRDC that may induce similar results, such as *M. hyopneumoniae* and swIAV. However, the primary consideration was the effect of immunosuppressive viruses (such as PRRSV, PCV2, and PRV) on bacteria. Thus, these pathogens were not included in the test indicators. Furthermore, PCR methods are more accurate than seroprevalence approaches in identifying current pathogens (5).

## Conclusion

In conclusion, PRRSV, PCV2, *S. suit*, and *G. parasuis* are the major pathogens causing PRDC in China, with mixed infection being highly common. The dominant epidemic serotypes of *S. suis, G. parasuis*, and *P. multocida* were serotype 2, serotype 1, and capsular serogroups D, respectively. The susceptibility of pigs of different ages to pathogens was different, e.g., the detection rates of PRRSV, PCV2, and *G. parasuis* were higher in nursery pigs than those of *P. multocida* and *A. pleuropneumoniae* in fattening pigs. PRRSV and PCV2 are considered the major pathogens of PRDC, strong positive correlations were found between coinfection by PRRSV and *G. parasuis*, PRRSV and *P. multocida*, PCV2 and *P. multocida*, and also by PRRSV-PCV2 and *G. parasuis* and PRRSV-PCV2 and *P. multocida*. Based on these findings, we recommend focusing on the role of both immunosuppressive viruses and bacterial pathogens in PRDC prevention and control.

## Data availability statement

The raw data supporting the conclusions of this article will be made available by the authors, without undue reservation.

## Ethics statement

Ethical review and approval was not required for the animal study because sample collection was done in the course of routine diagnostics on farm.

## Author contributions

QS and QH conceptualized and designed the study. QS, XY, and DH conducted experiments, data curation, and analysis. QS wrote the original manuscript. QS, DH, and QH revised the manuscript. QS, XK, and HZ worked on methodology and resources. QS, BH, and WZ worked on data review and visualization. QH was responsible for funding acquisition, project administration, and supervision. All authors contributed to the article and approved the submitted version.

## Funding

This work was supported by the China Agriculture Research System of MOF and MARA (No. CARS-35) and the DBN Public Welfare Fund for young scholars of HZAU (No. 99921001).

## Conflict of interest

Author HZ is employed by Wuhan Green Giant Agriculture, Agriculture and Animal Husbandry Co., Ltd, China. The remaining authors declare that the research was conducted in the absence of any commercial or financial relationships that could be construed as a potential conflict of interest.

## Publisher's note

All claims expressed in this article are solely those of the authors and do not necessarily represent those of their affiliated organizations, or those of the publisher, the editors and the reviewers. Any product that may be evaluated in this article, or claim that may be made by its manufacturer, is not guaranteed or endorsed by the publisher.
