# Supplementary material for: Investigation and analysis of etiology associated with porcine respiratory disease complex in China from 2017 to 2021
Source: Front Vet Sci. 2022 Oct 11;9:960033. doi: 10.3389/fvets.2022.960033 (PMC9592729; doi:10.3389/fvets.2022.960033)
Supplement: Supplementary file 1 [file Table_1.docx]

**Supplementary Table 1.** **PCR primers used in this study**

| Pathogens | Gene | Sequence (5’−3’) | Size (bp) | Source |
| --- | --- | --- | --- | --- |
| PCV2 | ORF2 | CACGGATATTGTAGTCCTGGT  CGCACCTTCGGATATACTGTC | 494 | This study |
| PRRSV | ORF7 | ATGGCCAGCCAGTCAATCA  TCGCCCTAATTGAATAGGTG | 389 (PRRSV-1)  434 (PRRSV-2) | This study |
| PRV | gE | TTTGGATCCATGCGGCCCTTTCTG  TTTGAATTCTTACGACACGGCGTCGCA | 366 | This study |
| *S. suis* | 16S rRNA | CAGTATTTACCGCATGGTAGAT  GTAAGATACCGTCAAGTGAGAA | 294 | (18) |
| *A. pleuropneumoniae* | *Apx* IV | GCTCACCAACGTTTGCTCAT  GGGGACGTAACTCGGTGATT | 377 | (19) |
| *G. parasuis* | 16S rRNA | GTGATGAGGAAGGGTGGTGT  GGCTCGTCACCCTCTGT | 821 | (20) |
| *B. bronchiseptica* | *f la* | GCTCCCAAGAGAGAAAGGCT  GGTGGCGCCTGCCCTATC | 235 | (21) |
| *P. multocida* | *kmt 1* | ATCCGCTATTACCCAGTGG  GCTGTAAACGAACTCGCCAC | 457 | (22) |
